# Supplementary material for: DMRforPairs: identifying Differentially Methylated Regions between unique samples using array based methylation profiles
Source: BMC Bioinformatics. 2014 May 15;15:141. doi: 10.1186/1471-2105-15-141 (PMC4046028; doi:10.1186/1471-2105-15-141)
Supplement: Additional file 2 — DMRforPairs output for the comparison of A431-MCF7 and NA17018-NA17105. Please start from the HTML files in each folder. Available via the BMC Bioinformatics website. [file 1471-2105-15-141-S2.zip › 1394847754114233_MOESM2_ESM/A431_MCF7/figures/3806.pdf]

RegionID: 3806, chr1:155043322−155043967−M_values

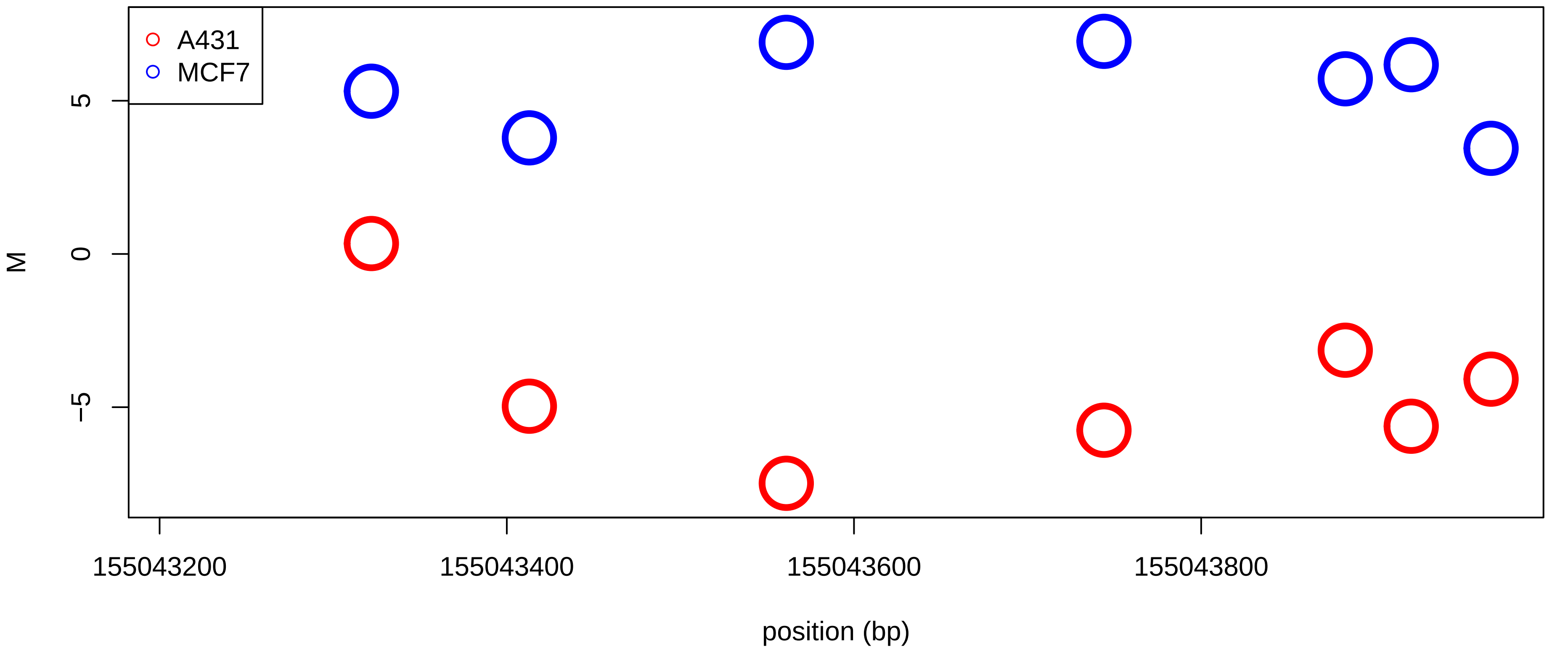

RegionID: 3806, chr1:155043322−155043967−Beta_values

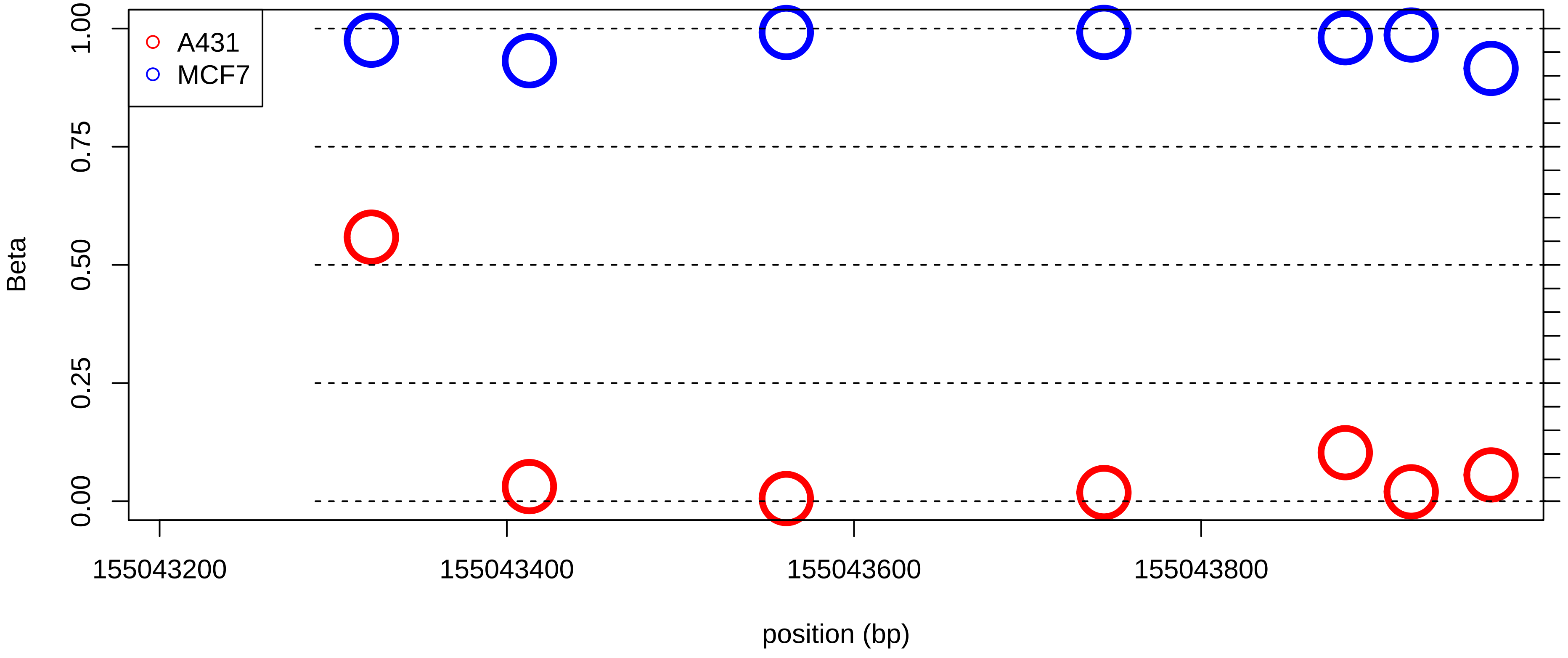

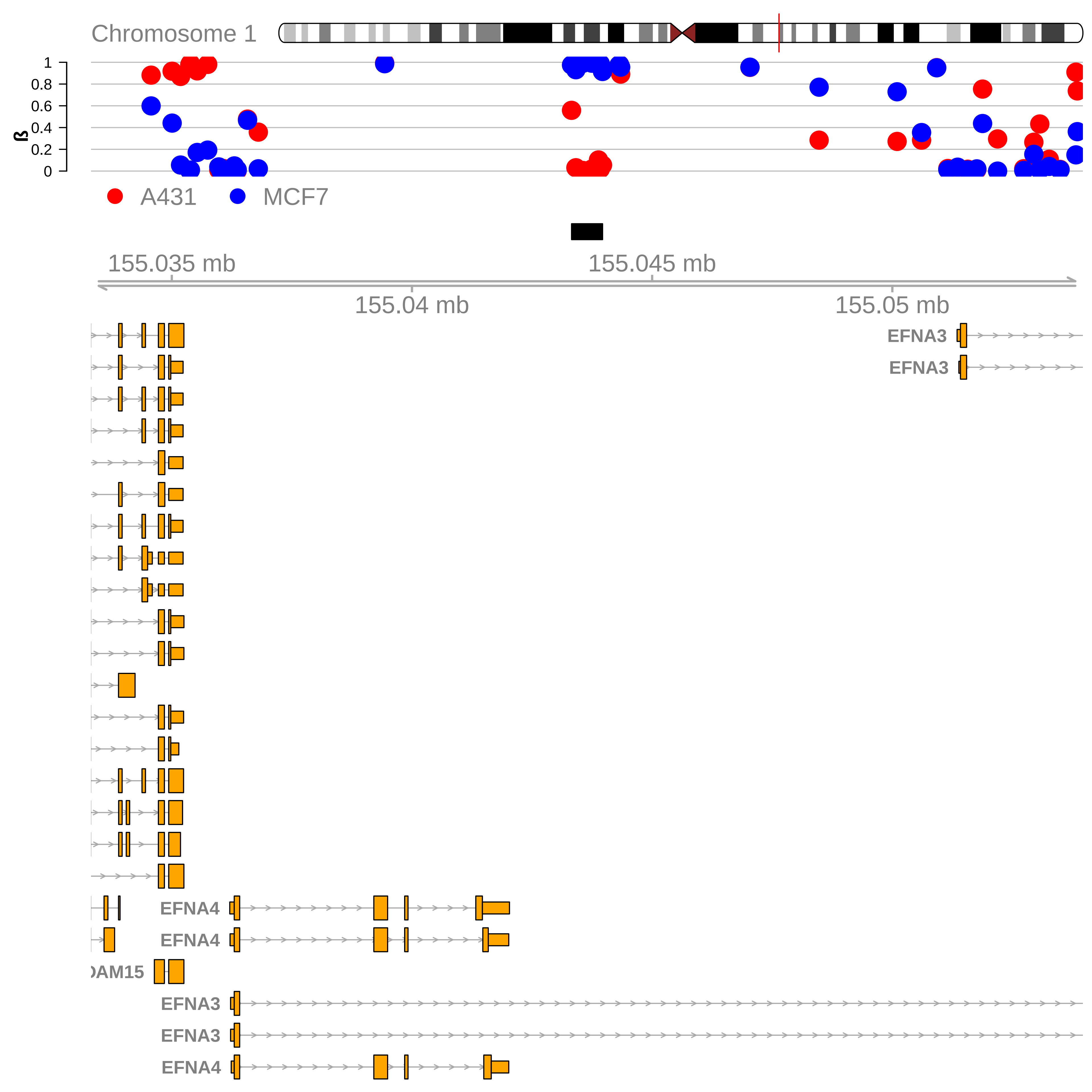

Chromosome 1
ß
1
0.8
0.6
0.4
0.2
0
A431
MCF7
155.035 mb
155.045 mb
155.04 mb
155.05 mb
EFNA3
EFNA3
EFNA4
EFNA4
EFNA3
EFNA3
EFNA4
